# Doing their best: strategies used by South African clinicians in working with psychiatric inpatients across a language barrier

**DOI:** 10.3402/gha.v8.28155

**Published:** 2015-10-26

**Authors:** Sanja Kilian, Leslie Swartz, Bonginkosi Chiliza

**Affiliations:** 1 Alan J Flisher Centre for Public Mental Health, Department of Psychology, Stellenbosch University, Stellenbosch, South Africa; 2 Department of Psychiatry, Stellenbosch University, Tygerberg, South Africa

**Keywords:** language barriers, lower income countries, psychiatric care, optimal clinical care, Roter interaction analysis system

## Abstract

**Background and objectives:**

South Africa has 11 official languages, but most psychiatrists can speak only English and Afrikaans and there are no formal interpreter posts in the mental healthcare system. As a result clinicians communicate with patients who have limited English language proficiency (LEP) without the use of interpreters. We present case material, constituting recordings of interactions between clinicians and LEP patients in a public psychiatric institution. The aim is to have a better understanding of how these clinical encounters operated and what communicative strategies clinicians used.

**Design:**

We used the Roter interaction analysis system (RIAS) to evaluate clinicians’ conversational strategies and to analyze interactions between clinicians and patients.

**Results:**

Clinicians showed a high degree of tenacity in trying to engage patients in the clinical conversation, build rapport, and gather crucial diagnostic information. However, patients often responded briefly and monosyllabically, or kept quiet. In psychiatry where commonality of language cannot be assumed, it is not possible to determine the clinical significance of these responses.

**Discussion:**

Clinicians went to great lengths to understand LEP patients. It is also clear that patients were often not optimally understood. Clinicians would try to gain valid information in a polite manner, but would abandon these attempts repeatedly as it became clear that proper communication was not possible.

**Conclusions:**

Our findings suggest that in the absence of interpreter services, the communication between clinicians and LEP patients is sparse and yields limited clinical information. The lack of proper language services stands in the way of optimal clinical care and requires urgent attention.

Language is at the heart of psychiatric practice. Proper diagnosis and management of mental disorder – even if the treatment is primarily biological – generally depend on the ability of the clinician to communicate with and understand the patient. Globally, though, it is not uncommon for clinicians not to be able to understand the languages spoken by patients, and vice versa (1), and there is now a sophisticated literature on how best to work with interpreters in mental healthcare settings in order to bridge language gaps. Most of this literature comes from high-income countries where resources are less constrained. However, less is known about language access to mental healthcare in low- and middle-income countries.

South Africa is an upper middle-income country and is linguistically diverse. There are 11 official languages and a constitutional commitment to the development of South African sign language. Despite this, the reality is that most psychiatrists and psychologists can speak only English and Afrikaans (the two official languages under apartheid), and there are no formal interpreter posts in the mental healthcare system. A number of studies have documented the challenges associated with this lack of provision, with most of them focusing on the use of informal, *ad hoc* interpreters such as hospital cleaners and security guards (2–6). One issue, which is clear from this work, is that mental healthcare facilities are very busy, and clinicians may feel reluctant to take staff such as cleaners and security guards away from their work to assist as informal interpreters. In such situations, clinicians may attempt as best they can to work with patients who have limited English language proficiency (LEP), without the use of informal interpreters. Despite the fact that this may be a common practice, we found no research exploring what occurs between clinicians and patients in such encounters.

As a first step to understand this situation, and as part of a larger study (7), we present here an analysis of a small corpus of case material, constituting recordings of interactions between clinicians and LEP patients in a public psychiatric institution in Cape Town. We were interested to know how these clinical encounters operated and what strategies clinicians used to try to understand and communicate with their patients. In all cases, the patients were asked whether they could speak English and in all cases they said that they could do so.

In the Western Cape, one of the nine provinces of South Africa, the majority of people are first language Xhosa-speakers (1 of the 11 official South African languages). Prior to 1994, the hospital in question served mainly Afrikaans- and English-speaking patients. After the fall of apartheid, the institution experienced, and continues to experience, an increase in Xhosa-speaking patients. However, at the time of our data collection, the hospital had very few Xhosa-speaking clinicians and did not employ official interpreters. The larger study focused mainly on conversations between clinicians and patients during psychiatric evaluations mediated by *ad hoc* interpreters and we report on this elsewhere (8). Given the resource challenges, however, some psychiatric evaluations were conducted without the use of an interpreter and we report here on these findings.

## Design

### Study design and setting

This particular hospital was chosen for the purpose of this study, due to historical reasons explained in the Introduction section. We used purposive and snowball sampling for this study. The first author approached a senior psychiatrist who is in charge of various wards at the hospital in question and explained the nature of the study. The senior psychiatrist put the first author in contact with clinicians (also known as registrars or psychiatrists in training) working under his supervision. The first author explained the study to clinicians and they agreed to participate. They also put the first author in contact with other clinicians (i.e. snowball sampling). During discussions, the clinicians decided that they would contact the first author whenever they have psychiatric interviews (also referred to as consultations) scheduled with patients who are not first language English speakers. Once notified, the first author video-recorded the interviews. Clinicians were therefore responsible for recruiting patients and were first to obtain consent from patients. Thereafter, the first author also approached the patients and again explained the nature of the study and obtained consent. None of the registrars or patients refused to participate.

The first author was personally responsible for making video-recordings of the psychiatric interviews and was present during the recordings and made observations and field notes. Over the course of approximately 6 months, the first author video-recorded 25 real-life psychiatric interviews. Seven of the real-life psychiatric interviews were dyadic and did not involve an interpreter as clinicians were informed by nursing staff that these patients were able to communicate in English. The clinicians were first language English or Afrikaans speakers and patients were first language isiXhosa speakers. All the patients were inpatients and male and all the registrars were female. Unfortunately, we do not have information pertaining to participants’ age and educational status. The study was approved (N09/05/162) by the Committee for Human Research at Stellenbosch University, as well as by the board of the hospital.

### Analysis

We used the Roter interaction analysis system (RIAS) (9) to evaluate clinicians’ conversational strategies in working in English with LEP patients. RIAS is a tool specifically designed to describe and summarize communication in clinical settings and provides a frequency count and estimation of the prominence of the conversational strategies used throughout a conversation (9).

It codes dialog between patients and clinicians according to various communicative tasks that fall into broader categories. The categories are focused on gathering data (Data-gathering – Biomedical; Data-gathering – Lifestyle/psychosocial); providing information (Patient Education and Counseling – Biomedical, Patient Education and Counseling – Lifestyle/psychosocial); facilitating the conversation and engaging the patient (Facilitation and Patient Activation); building rapport (Rapport-building – Positive, Rapport-building – Emotional, Rapport-building – Negative, and Rapport-building – Social); and creating conversational flow (Procedural). For example, when a clinician paraphrases what the patient said this task is coded under the category Facilitation and Patient Activation. The RIAS tool provides a frequency count of the number of times this category occurred throughout a particular conversation and across multiple conversations. It also provides an estimation (percentage) of the proportion of the clinician's talk allocated to a particular category. In order to minimize bias, the transcribed interviews were scored not by ourselves but by the RIAS team in the United States. The authors, informed by field notes made by the first author, reviewed the RIAS coding and interpreted the results.

## Results

In the first part of this section, we provide a summary of the communicative strategies (categories and communicative tasks) that clinicians used to communicate with LEP patients across the seven psychiatric interviews (see Table 1). Thereafter, we provide examples of the communicative strategies employed.

**Table 1:** Summary of clinicians’ communicative strategies

Communication category	Communicative tasks	%	Rank
Facilitation and Patient Activation	Asks for opinion; asks for permission; asks for reassurance; asks for understanding; back-channels; paraphrase, checks for understanding	30.2	1
Data-gathering Lifestyle/psychosocial	Closed question – lifestyle; closed question – psychosocial; open question – lifestyle; open question – psychosocial	27.3	2
Rapport-building – Positive	Laughs, tells jokes; approval – direct; compliment – general; shows agreement, understanding	11	3
Rapport-building – Emotional	Empathy statements; legitimizing statements; concern, worry; reassurance, optimism; partnership statements; self-disclosure.	10.3	4
Data-gathering – Biomedical	Closed question – medical, closed question – therapeutic, closed question – others; open question – medical; open question – therapeutic; open question – others; bid for repetition	7.6	5
Patient Education and Counseling – Lifestyle/psychosocial	Gives information – lifestyle; gives information – psychosocial; counsels – lifestyle/psychosocial	5.6	6
Patient Education and Counseling – Biomedical	Gives information – medical; gives information – therapeutic; gives information – others; counsels – medical/therapeutic	4.7	7
Procedural	Transitions; gives orientation, instructions	2.3	8
Rapport-building – Negative	Disagreement, criticism – direct; disagreement, criticism – general	0.5	9
Rapport-building – Social	Personal remarks	0.5	9

### Summary of communicative tasks and categories

Table 1 provides a summary of the different communicative strategies used by clinicians throughout the entire corpus of data. The percentage and rank provided in the table is an estimate of the proportion of talk allocated to a particular category and the prominence thereof.

### Examples of clinicians’ communicative strategies

Clinicians’ efforts during the psychiatric evaluations were mainly centered on Facilitation and Patient Activation (30.2%) – this was most prominent in the clinicians’ talk across the evaluations. This category in particular speaks to the idea that clinical conversations should be a partnership (9). It should not be talk between the clinician, who is the expert and therefore has all the power, and the voiceless patient who has no power (9). In recent times, healthcare practitioners have become more focused on patient centeredness and more critical of their own communicative behaviors (10).

The following examples illustrate clinicians’ attempts to facilitate conversation and engage patients throughout clinical encounters. In Extract 1, the patient explains that he feels stiff and that this bothers him. The clinician checks for understanding and tries to establish whether the patient has any other concerns in addition to feeling stiff.

Extract 1:

Patient: I'm feeling not right. I don't feel right, but I'm not ease because I'm stiff.

Clinician: Mm?

Patient: I'm not feeling happy, that because I'm still stiff.

Clinician: So you're still feeling a bit stiff?

Patient: Yes.

Clinician: Ok, but is that the only reason you are not happy or are there other reasons that you're not happy?

Patient: yes.

Clinician: What are the other reasons?

No response from patient.

Clinician: Or is it just the stiffness that's bothering you?

No response from patient.

Clinician: Is there anything else bothering you?

Patient: No.

In Extract 2, the clinician asks for reassurance by repeating the same question relating to the patient's sleeping habits.

Extract 2:

Clinician: Are you having difficulty sleeping every night?

Patient: Yes.

Clinician: Or just last night or some nights?

Patient: Some nights.

Clinician: Some nights.

Patient: Some nights.

The category Facilitation and Patient Activation also refers to asking for permission and patients’ opinions regarding important matters. In Extract 3, the clinician, in relation to a reference the patient made about finding work as a security guard, asks the patient's permission to find someone from the hospital to assist him in finding employment.

Extract 3:

Patient: My plan is to get a work, a security work.

Clinician: Oh, ok. Have you ever been a security guard before?

No response from patient.

Clinician: Have you worked in security in the past?

Patient: No, I have not …

Clinician: Ok, do you want to do training?

Patient: Yes, I want to do training.

Clinician: Um, perhaps I can ask, we have a lady here that helps when, with people, organizing for work. And who can give you some help maybe.

No response from patient.

Clinician: Would you like me to ask maybe ask her to talk to you?

Patient: Yes.

In Extract 4, the clinician tries to get the patient's permission and to engage the patient in an important decision about rehabilitation. See below for the dialog that arose between the clinician and patient.

Extract 4:

Clinician: I'm going to speak to the social worker, she's your social worker. And we'll see if we can try and organise something for the rehab centres where you go and stay for a few weeks, normally there is a waiting list. Is that something you would be interested in? Would you go and stay somewhere for a while?

No response from patient.

Clinician: Or would you rather go to outpatients? Where you are at home, but you go to the group once a week and then you go back home?

No response from patient.

Clinician: Which one would you prefer?

Patient: So, I must go. First I go?

Clinician: But I think it's important, it's something that you need to do for yourself, because you want to make (inaudible). It's not something I can send you to do, or make you do it. It's something that you need to want to change.

No response from patient.

Clinician: It's something that you need to want to do and then it can work really well.

No response from patient.

In another example (see Extract 5), the clinician tries to engage the patient in making decisions about changing from oral to injectable antipsychotic treatment. The patient's participation in this decision is crucial since it will have an impact on the patient's life. Many patients feel that injectable medication takes away the power they have in managing their illness and this could affect adherence, while other patients prefer injectable medication and find it to be life-changing.

Extract 5:

Clinician: Promise yourself. It's awful to take pills, we're going to see if we can get you on injections only. Would you prefer that, I think maybe it's easier for you if you just have the injection every month at the clinic.

No response from patient.

Clinician: We'll see if we can organize something where you don't have to take so many pills and have the injection.

No response from patient.

Patient: Ok.

Clinician: Ok. But we will talk about that again.

It is clear from the examples of clinicians’ strategies focused on Facilitation and Patient Activation that these attempts did not have the desired effect, regardless of clinicians high degree of tenacity in trying to communicate and engage patients. It seems that patients, to a great extent, remained voiceless during clinical conversations.

The second most prominent category in clinicians’ talk was Data-gathering – Lifestyle/psychosocial (27.3%). The prominence of this is not unusual since this information is essential to make a psychiatric diagnosis and to monitor patients’ progress. Behavior categorized under this category refers to questions about patients’ family and home life and more importantly psychotic symptoms. For example, in Extract 6, the clinician asks the patient about the nature of the voices bothering him. Again, the patient does not offer detailed information pertaining to his symptoms.

Extract 6:

Clinician: The voices are still bothering you?

Patient: Yes.

Clinician: Ok, what are they saying?

Patient: They are saying, I'm not right.

Clinician: Ok, are they saying anything else?

Patient: Yes, they are saying just like that, I'm not right.

In another example, the clinician tries to gather diagnostic information about the patient's delusions and verbal hallucinations. The patient provides short responses and at one point he is unresponsive, leaving the clinician with sparse clinical information.

Extract 7:

Clinician: That feeling that you had before, about Jesus, that you were going to be Jesus, do you still have that feeling?

Patient: No.

Clinician: Has it gone away completely, totally gone?

Patient: Yes, completely gone.

Clinician: Do you have any other special powers or abilities? Are there any things that you can do, that other people can't do, that you want to tell me about?

No immediate response from patient.

Clinician: I mean you told me that you had this feeling that you could be Jesus or a prophet. Is there any other feeling that you have?

Patient: No.

Clinician: No other feelings. Any other ideas like, are you hearing any voices that other people can't hear?

Patient: No.

Clinician: Have you ever heard the voices?

Patient: I have before.

Clinician: And at the moment?

Patient: No.

Clinician: Have you heard the voices at all this week?

Patient: No.

Clinician: Ok, so the voices have gone?

Patient: Have gone.

Clinician: And the Jesus prophet feeling has gone?

Patient: Yes, have gone.

At this point, the clinician abandons this line of enquiry but it is by no means clear what the patient has understood and attempted to communicate.

The process of gathering reliable clinical information pertaining to psychotic symptoms is further complicated by the fact that patients’ responses (commonly very short to the point of being monosyllabic) could also be a product of the patients’ illness, or of lack of understanding of illness, or of other factors (such as not wishing to take the clinician's advice but not wishing to engage in open conflict), or a combination of all of these.

The third most prominent category in clinicians’ talk was Rapport-building – Positive (11%). This category involves giving direct approval, making general compliments and showing agreement or understanding and was followed by the category Rapport-building – Emotional (10.3%). The latter includes empathy statements; legitimizing statements; concern; reassurance or optimism; partnership statements; and self-disclosure. In Extract 8, the clinician, in the same turn, used rapport-building positive and emotional. The clinician compliments the patient on his progress and also expresses her concern over the patient's drug abuse.

Extract 8:

Clinician: Are there any questions you want to ask me?

Patient: No questions I want to ask.

Clinician: Ok, well I think things are going quite well. I can see you've made an improvement, since you came to the hospital. One of the things that really worry me is your continuing use of the drugs.

Patient: I never use the drug again. I promise doctor.

Clinician: I think the drugs are causing you a lot of problems with your brain.

Patient: Yes.

Clinician: They are damaging your brain.

Patient: Yes.

Clinician: And I think one of the problems you must understand is that each time you come into the hospital for a long time and then you go and you don't take the medicine and then you come back again. You can get worse each time and perhaps never return to the way you used to be. So it's really important that you do understand that the drugs do damage the brain.

No response from patient.

Clinician: It's extremely serious. And it's your responsibility to yourself and to your family. You know you say you want a career in security, those things will be possible for you, I'm sure. But if you continue using the drugs, it means you will be readmitted to hospital again and again. And if you steal money to get drugs you will end up in prison. So you have to really.

No response from patient.

Categories that occurred less frequently in clinicians’ talk were: Data-gathering – Biomedical data (7.6%); Patient Education and Counseling – Lifestyle/psychosocial (5.6%); Patient Education and Counseling – Biomedical (4.7%); Procedural (2.3%); Rapport-building – Negative (0.5%); and Rapport-building – Social (0.5%). In terms of gathering biomedical information, clinicians asked patients questions pertaining to physical symptoms they were experiencing due to drug-related side effects, appetite, sleep patterns, and questions about patients’ HIV status and tuberculosis (TB) symptoms. Tasks centered on patient education counseling involved giving mainly psycho-educational information. For example, one of the clinicians explained what rehabilitation entails: ‘… it's a concept where you learn how to stop using drugs and how to live a life without drugs. And the substances, the drugs you are using, are very, tik (also known as methamphetamine) is very, very addictive and your body craves it and want it. It's a very difficult thing stop alone. But if you are in a group with other people there are a lot more support there from other people. Who also have the same problems as you and you can support each other. And it's a system that works very well, where you can be with other people who also try to stop using drugs. And there is also a medium, where a counselor, somebody who can give you advice and talk through the reasons why we use drugs. Why you want to use drugs, but why you shouldn't use them’. The type of information clinicians gave about biomedical issues related to medication type and side effects associated with antipsychotic medication. The communicative category Rapport-building – Negative involves disagreeing with patients and in Extract 9, the clinician alludes to the disagreement between the patient and clinician about the patient having a mental illness.

Extract 9:

Clinician: Ok. Um, look Nomakephu, um I know that you don't think this is true, but um, we, we know that you have a mental illness.

Patient: Yes.

Clinician: Ok, we think that some of the things that you are experiencing …

Patient: Yes.

Clinician: Are because of a problem with your mind.

Patient: Yes.

Clinician: Ok, and definitely dagga (cannabis) can cause some of that problem with your mind. So that is why we always tell you that you mustn't smoke dagga, ok.

Patient: Ok.

Clinician: Ok, and when we start to feel that you are getting a bit better we can discuss again ways that can get you off the dagga, if that is what you choose to do. But you are here because you are sick and we need to give you some medications that will help you to get better.

Patient: Ok.

Clinician: And some of the things that are happening to you will then stop happening. And last time, you know, the medications didn't work as well as we liked and that's why you had to come back, ok.

Patient: Yes.

Rapport-building – Social involves giving personal remarks. Finally, the category Procedural refers to giving orientation and establishing a flow in the dialog. In our findings, clinicians, as can be expected, were responsible for moving the conversation into a new direction by introducing a new topic. For example, clinicians often asked in the first part of the consultation about substance dependency and directly thereafter introduced questions relating to clinical symptoms. This makes sense in a psychiatric interview, since information related to substance dependence will inform questions about clinical symptoms.

## Discussion

The data confirm clinical and research experiences in similar settings over 30 years (11–13). Most noteworthy from the data were the repeated attempts by clinicians to engage with and communicate clearly with patients. According to the RIAS scoring system, which was independently rated, the clinicians appropriately focused on rapport-building and data-gathering activities, and the qualitative data illustrate the lengths they went to understand patients.

It is also clear from the data that patients were often not optimally understood and that they responded very briefly and at times monosyllabically, and at other times simply kept quiet. In a situation in which commonality of language cannot be assumed, it is not possible to determine the psychiatric significance of these responses. Clinicians would try to gain valid information in a polite manner, but would abandon these attempts repeatedly as it became clear that proper communication was not possible.

These findings are consistent with those of Steyn (14) who heard from clinicians working in settings such as this one of their ongoing frustrations at not being able to communicate with patients. Overlaid with these experiences, however, was a feeling from these clinicians that they were doing something wrong – that if they were more skilled communicators, they would have learned more from patients. Our data suggest that the problem lies not with underdeveloped clinical skills but with the broader situation in which there is not adequate provision of accessible language services.

The task clinicians undertake in these circumstances appears to us to be almost impossible. What is being played out at the clinical level – and at a level which profoundly affects the lives of patients – is a product of ongoing lack of provision in a country in which, in terms of the constitution, there should be equal access to healthcare and non-discrimination on the basis of language.

In our larger study, we are working with and following up a cohort of community interpreters who are now working in the healthcare system with support and supervision from our group. This pilot project will give information about low-cost language interventions. There are, however, broader issues at stake – patients with mental disorder in public institutions in South Africa are among the most excluded and marginalized in the country, and ongoing activism on this issue is essential. Our hope is that the corpus of data in this article, though small, will assist in illuminating a problem very familiar in mental healthcare in South Africa, but rendered invisible through its routinization and normalization as part of the everyday challenges of working in our context (15). This is in keeping with the call by Bhui (16) for the use of qualitative research to inform new interventions and developments in culturally appropriate mental healthcare.

### Strengths of the study

As far as we know, this is the first attempt made to analyze data between clinicians and LEP patients in public psychiatric care in Africa.

### Limitations of the study

The evidence presented in this paper consists of only seven psychiatric interviews collected in one particular psychiatric hospital. Our study findings are therefore not generalizable. Nevertheless, our study may help clinicians better communicate with LEP patients in the absence of interpreters.
